# Comprehensive Analysis of the Oncogenic Role of Targeting Protein for Xklp2 (TPX2) in Human Malignancies

**DOI:** 10.1155/2022/7571066

**Published:** 2022-10-18

**Authors:** Ting Shao, Xiling Jiang, Guochang Bao, Chunsheng Li, Changgang Guo

**Affiliations:** ^1^Department of Gynecology, Affiliated Hospital of Chifeng University, Chifeng 024000, China; ^2^Department of Stomatology, Affiliated Hospital of Chifeng University, Chifeng 024000, China; ^3^Inner Mongolia Key Laboratory of Oral Craniofacial Diseases, Chifeng University, Chifeng 024000, China; ^4^Department of Urology, Affiliated Hospital of Chifeng University, Chifeng 024000, China; ^5^Urology Research Center, Chifeng University, Chifeng 024000, China

## Abstract

Mitosis and spindle assembly require the microtubule-associated protein *Xenopus* kinesin-like protein 2 (TPX2). Although TPX2 is highly expressed in several malignant tumor forms, little is known about its role in cancer. In this study, we performed the gene set enrichment analysis of TPX2 in 33 types of cancers and an extensive pan-cancer bioinformatic analysis using prognosis, tumor mutational burdens, microsatellite instability, tumor microenvironment, and immune cell infiltration data. According to the differential expression study, TPX2 was found to be overexpressed across all studied cancer types. Based on the survival analysis, increased TPX2 expression was associated with a poor prognosis for most cancers. The TPX2 expression level was confirmed to correlate with the clinical stage, microsatellite instability, and tumor mutational burden across all cancer types. Furthermore, TPX2 expression has been linked to tumor microenvironments and immune cell infiltration, particularly in bladder urothelial carcinoma, liver hepatocellular carcinoma, lung adenocarcinoma, stomach adenocarcinoma, and uterine corpus endometrial carcinoma. Finally, the gene set enrichment analysis implicated TPX2 in the regulation of aminoacyl tRNA biosynthesis, which is the most important tumor cell cycle signaling pathway. This comprehensive pan-cancer analysis shows that TPX2 is a prognostic molecular biomarker for most cancers and suggests its potential as an effective therapeutic target for the treatment of these diseases.

## 1. Introduction

Owing to its high expression throughout the cell cycle, p100, now known as human TPX2 (targeting protein for Xklp2) [1, 2], was first identified as a microtubule-associated protein (MAP) responsible for mediating the localization of the kinesin-like protein Xklp2 to the ends of microtubules during mitosis [3, 4]. It is a microtubule-associated protein required for the development and function of mitotic spindles [5]. TPX2 possesses a nuclear localization sequence and is localized to the nucleus during interphase; further, it localizes to spindle microtubules during mitosis, with a preference for the spindle poles [6]. Like spindle flypaper, TPX2 directs numerous proteins to the spindle. For example, TPX2 binds the mitotic kinase Aurora A, activates it, and localizes it to the spindle [7]. In the vicinity of chromosomes, the chromatin-driven spindle assembly pathway exerts such regulatory effects, and TPX2 is one of the main targets of this pathway [8]. Microtubule-binding proteins, motors, and nucleation factors are only few of the proteins that TPX2 interacts with directly or indirectly to regulate spindle formation and activity [9]. The role of TPX2 in tumor metabolism and tumor immunity has not yet been discovered [10].

High-throughput sequencing methods and next-generation omics platforms have enabled the unparalleled molecular profiling of various diseases in recent years [11]. Transcriptome sequencing has become the primary tool for measuring gene expression owing to technological advancements and its lower costs [12]. Furthermore, the remarkable bioinformatic revolution has led to the generation of a vast quantity of data that could be utilized to map out information about all known genes and cancer types and identify useful patterns [13]. Pan-cancer analysis is a bioinformatic approach that uses data from multiple databases to assess the expression, mutational pattern, and function of a gene in the context of various cancers, taking into consideration patient prognoses [14]. Such analyses can provide insight into the function of the genes involved and their interactions in different types of cancers.

Whereas TPX2 overexpression has been observed in a variety of cancers, no thorough pan-cancer investigation of TPX2 has previously been performed. Our study is aimed at elucidating the role of TPX2 in cancer metabolism and immunity by performing a pan-cancer analysis of integrated multiomics data. In this study, bioinformatic analysis was performed to investigate the relationship among TPX2 expression, prognosis, clinical stage, tumor mutational burden (TMB), microsatellite instability (MSI), tumor microenvironment, and immune cell infiltration (ICI) in multiple cancers.

## 2. Materials and Methods

### 2.1. Raw Data and TPX2 Expression Analysis

Gene expression RNA-sequencing data (HTSeq-FPKM), somatic mutation data (VarScan2 variant aggregation and masking), and clinical data for 33 cancer types were retrieved from The Cancer Genome Atlas (TCGA) database and downloaded using the UCSC Xena (https://xena.ucsc.edu/) [15]. For the 33 cancer types (Table S1), a boxplot was constructed using the R package ggpubr to show differences in TPX2 expression between cancerous and normal tissues. The statistical method to analyze differences was the Wilcoxon signed-rank test.

### 2.2. Prognostic Survival Analysis

To learn more about the prognostic significance of TPX2 in various cancers, we evaluated the connection between TPX2 expression and patient survival using data from TCGA database. In different cancer types, we evaluated the relationship between TPX2 expression levels and overall survival (OS), disease-free survival (DFS), progression-free survival (PFS), and disease-specific survival (DSS) using the Kaplan–Meier method and univariate Cox proportional-hazards analysis. Analysis and visualization were performed using the R packages limma, survival, survminer, and forestplot.

### 2.3. Correlation Analysis of TPX2 Expression with Clinical Stage, TMB, and MSI

TCGA database provides clinical-stage data for 33 types of cancers. An ANOVA with an LSD (least significance difference) post hoc test and the R packages limma and ggpubr were used to study the correlation between TPX2 expression and the clinical stage. When comparing two clinical stages, *p* < 0.05 was considered as statistically significant. TMB and MSI data for 33 types of cancers were obtained from TCGA database. TMB, defined as the total number of somatic mutations per coding region of a tumor genome [16], is a novel clinical biomarker linked to ICI therapeutic effectiveness. Insufficient repair of insertion-deletion loop mismatches, which occur during DNA replication in tandem repeat sequences across the genome, causes MSI, which has been linked to the development of a variety of cancers [17]. The correlation between TPX2 expression and TMB/MSI was analyzed for the 33 types of cancers using R software and the Spearman correlation method. Visualization was performed using the R package fmsb.

### 2.4. Evaluation of Tumor Microenvironments and ICI

The ImmuneScore, StromalScore, and ESTIMATEScore were calculated to predict tumor purity in the tumor microenvironment for each sample using the R packages limma and estimate. The correlation between TPX2 expression and the stromal score/immune score was analyzed in the tumor microenvironment using the Spearman correlation method and the R packages ggpubr and ggExtra. If *p* < 0.001, visualization was performed using the R package ggplot. Thorsson et al. [18], who used CIBERSORT (cell-type identification by estimating relative subsets of RNA transcripts), an analytical tool that imputes gene expression profiles and provides an estimation of the abundances of member cell types in a mixed cell population using gene expression data to construct pan-cancer immune cell infiltration score of TCGA database, provided us with these scores. Using the Spearman correlation analysis and the R packages ggpubr and ggExtra, the relationship between TPX2 expression and the number of infiltrating immune cells was investigated. Visualization was performed using the R package ggplot2. We also analyzed the correlation between TPX2 expression and immune checkpoint molecules using correlation test analysis and the R package limma, and the R packages reshape2 and the RColorBrewer were used to create the heat map.

### 2.5. Gene Set Enrichment Analysis (GSEA)

GSEA is a statistical approach for determining the expression status of genes inside a functional gene set by comparing them to predefined gene sets [19]. To elucidate the biological functions and pathways with which TPX2 is involved, we used the R packages limma, http://org.Hs.eg.db, clusterProfiler, and enrichplot to run GSEA on TPX2. We utilized the molecular signatures database (MSigDB), which includes all Gene Ontology (GO) and Kyoto Encyclopedia of Genes and Genomes (KEGG) gene sets. GSEA findings were considered significantly enriched with |NES| > 1 and nominal *p* < 0.05.

## 3. Results

### 3.1. TPX2 Expression across Cancer Types

**Figure 1:**
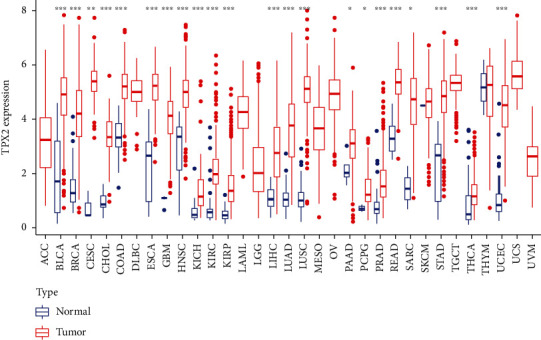
Boxplots depicting TPX2 expression differences between cancerous and normal tissues among 33 cancer types. ^∗^represents *p* < 0.05, ^∗∗^represents *p* < 0.01, and ^∗∗∗^represents *p* < 0.001.

TPX2 expression levels in cancerous tissues were much higher than those in normal tissues in 22 types of cancers, including BLCA, BRCA, CESC, CHOL, COAD, ESCA, GBM, HNSC, KICH, KIRC, KIRP, LIHC, LUAD, LUSC, PAAD, PCPG, PRAD, READ, SARC, STAD, THCA, and UCEC (Figure 1). There was no significant difference in TPX2 expression between cancerous and normal tissues for THYM and SKCM cancers. TCGA database lacks data for normal tissue samples for comparisons with cancers such as ACC, DLBC, LAML, LGG, MESO, OV, TGCT, UCS, and UVM. Among the 33 cancer types, UCS had the highest TPX2 expression, whereas KICH had the lowest.

### 3.2. TPX2 Is a Prognostic Biomarker for Several Cancer Types

**Figure 2:**
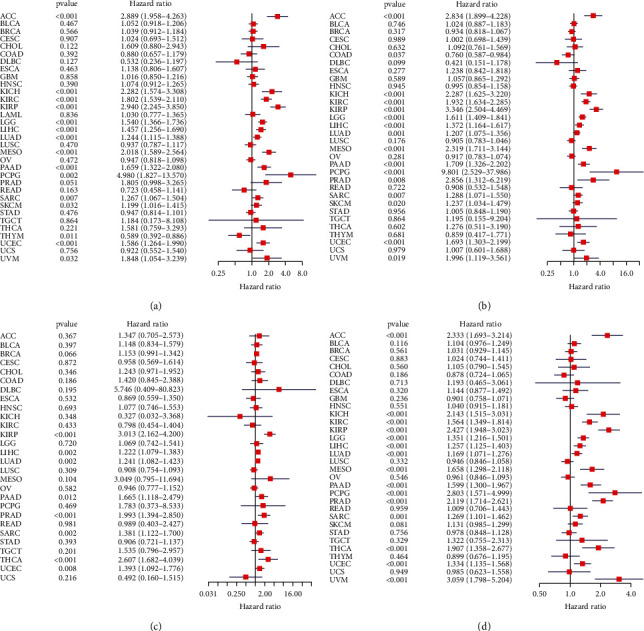
Univariate Cox proportional-hazards analyses comparing TPX2 expression levels and survival outcomes. Forest plots of hazard ratios between TPX2 expression levels and (a) overall survival, (b) disease-specific survival, (c) disease-free survival, and (d) progression-free survival in 33 cancer types.

Hazard ratios (HRs) were significant between TPX2 expression and OS for ACC (HR = 2.889), KICH (HR = 2.282), KIRC (HR = 2.282), KIRP (HR = 2.940), LGG (HR = 1.540), LIHC (HR = 1.457), LUAD (HR = 1.244), MESO (HR = 2.018), PAAD (HR = 1.659), PCPG (HR = 4.980), SARC (HR = 1.267), SKCM (HR = 1.199), THYM (HR = 0.589), UCEC (HR = 1.586), and UVM (HR = 1.848) (Figure 2(a)). HRs were also significant between TPX2 expression and DSS for ACC (HR = 2.834), COAD (HR = 0.760), KICH (HR = 2.287), KIRC (HR = 1.932), KIRP (HR = 3.346), LGG (HR = 1.611), LIHC (HR = 1.372), LUAD (HR = 1.207), MESO (HR = 2.319), PAAD (HR = 1.709), PCPG (HR = 9.801), PRAD (HR = 2.856), SARC (HR = 1.288), SKCM (HR = 1.237), UCEC (HR = 1.693), and UVM (HR = 1.996) (Figure 2(b)). Moreover, HRs were significant between TPX2 expression and DFS for KIRP (HR = 3.031), LIHC (HR = 1.222), LUAD (HR = 1.241), PAAD (HR = 1.665), PRAD (HR = 1.993), SARC (HR = 1.381), THCA (HR = 2.607), and UCEC (HR = 1.393) (Figure 2(c)). Finally, HRs were significant between TPX2 expression and PFS for ACC (HR = 2.333), KICH (HR = 2.143), KIRC (HR = 1.564), KIRP (HR = 2.427), LGG (HR = 1.351), LIHC (HR = 1.257), LUAD (HR = 1.169), MESO (HR = 1.658), PAAD (HR = 1.599), PCPG (HR = 2.803), PRAD (HR = 2.119), SARC (HR = 1.269), THCA (HR = 1.907), UCEC (HR = 1.334), and UVM (HR = 3.059) (Figure 2(d)).

**Figure 3:**
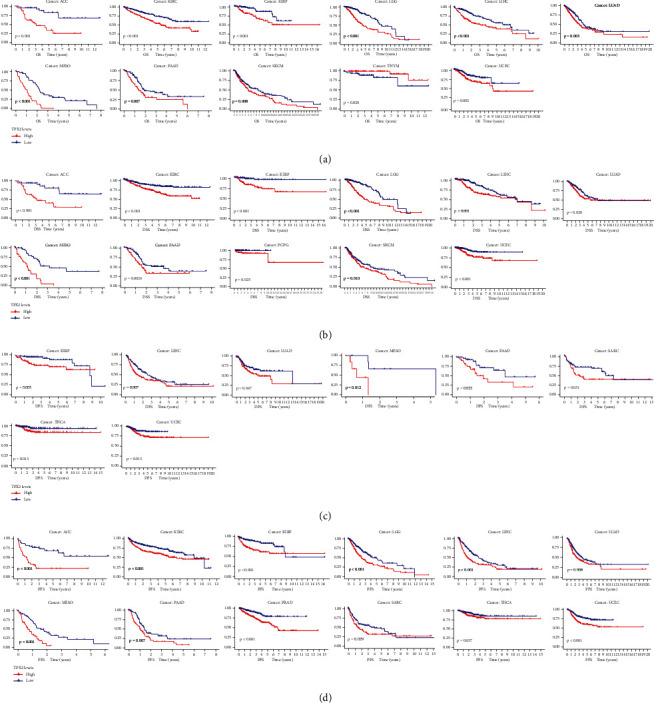
Kaplan–Meier survival analysis of patients with high and low TPX2 expression in numerous cancers. (a) Overall survival. (b) Disease-specific survival. (c) Disease-free survival. (d) Progression-free survival.*p* < 0.05 represents a significant difference between survival outcomes.

As shown in Figure 3, TPX2 expression was found to significantly affect prognosis, including OS, DSS, DFS, and PFS. Higher TPX2 expression indicated low OS in ACC, KIRC, KIRP, LGG, LIHC, LUAD, MESO, PAAD, SKCM, and UCEC but better OS in THYM (Figure 3(a)). Patients with TPX2 overexpression had lower DSS for ACC, KIRC, KIRP, LGG, LIHC, LUAD, MESO, PAAD, PCPG, SKCM, and UCEC (Figure 3(b)). Further, patients with high expression of TPX2 had lower DFS for KIRP, LIHC, LUAD, MESO, PAAD, SARC, THCA, and UCEC (Figure 3(c)). In ACC, KIRC, KIRP, LGG, LIHC, LUAD, MESO, PAAD, PRAD, SARC, THCA, and UCEC, patients with high TPX2 expression experienced shorter PFS (Figure 3(d)). The results thus showed that TPX2 expression in KIRP, LIHC, LUAD, PAAD, and UCEC is significantly correlated with OS, DSS, DFS, and PFS outcomes.

### 3.3. TPX2 Expression Is Associated with Clinical Data across Cancer Types

**Figure 4:**
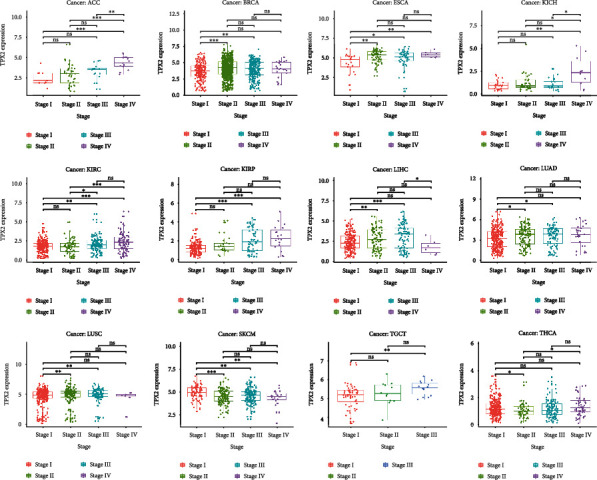
Correlation analysis between TPX2 expression levels and clinical T stage in 33 cancer types, showing the 12 cancer types for which there was a statistically significant difference. ^∗^represents *p* < 0.05, ^∗∗^represents *p* < 0.01, and ^∗∗∗^represents *p* < 0.001; and ns: not significant.

We next investigated the relationship between clinical data and TPX2 expression in 33 cancers.  Figure 4 displays the correlation between TPX2 expression levels and specific T classifications of the TNM staging system in ACC, BRCA, ESCA, KICH, KIRC, KIRP, LIHC, LUAD, LUSC, SKCM, TGCT, and THCA. Among them, especially for ACC, KICH, KIRC, KIRP, and TGCT, we found that the expression levels of TPX2 in most tumors increased with each increment of the T classification. These findings suggest that increased TPX2 expression levels are associated with cancer invasion and metastasis.

### 3.4. TPX2 Expression Correlates with TMB and MSI across Cancer Types

**Figure 5:**
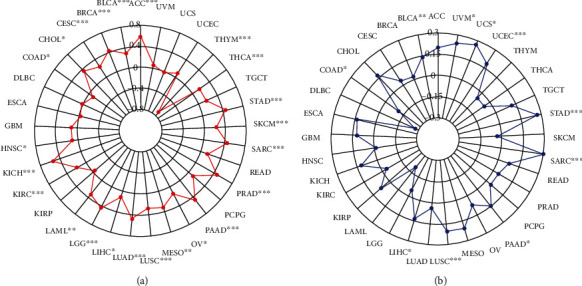
Correlation analysis of TPX2 expression levels with the tumor mutational burden (TMB) and microsatellite instability (MSI) across cancer types. (a) Results of correlation analysis between TPX2 expression levels and TMB. (b) Results of correlation analysis betweenTPX2 expression levels and MSI. ^∗^represents *p* < 0.05, ^∗∗^represents *p* < 0.01, and ^∗∗∗^represents *p* < 0.001.

In a variety of tumors, TMB and MSI are reliable prognostic biomarkers and indicators of immunological therapy responses. We evaluated their respective associations with TPX2 expression in various cancer types to uncover any link between TPX2 activity and mutations in certain cancer types. In 23 of the 33 cancer types for which data were available (ACC, BLCA, BRCA, CESC, CHOL, COAD, HNSC, KICH, KIRC, LAML, LGG, LIHC, LUAD, LUSC, MESO, OV, PAAD, PRAD, SARC, SKCM, STAD, THCA, and THYM), the relationship between TPX2 expression and TMB was significant (*p* < 0.05), and of these, THYM had the highest correlation coefficient (−0.7143, negative correlation), whereas COAD had the lowest correlation coefficient (−0.1023, negative correlation) (Figure 5(a)). The link between TPX2 expression and MSI was also investigated in 33 cancer types, with statistical differences found for BLCA, COAD, LIHC, LUSC, PAAD, SARC, STAD, UCEC, UCS, and UVM (Figure 5(b)). Among the several types of cancer, SARC had the highest correlation coefficient (0.2947, positive correlation), whereas COAD had the lowest correlation coefficient (−0.1090, negative correlation).

### 3.5. Tumor Microenvironment and Immune Cell Infiltration

**Figure 6:**
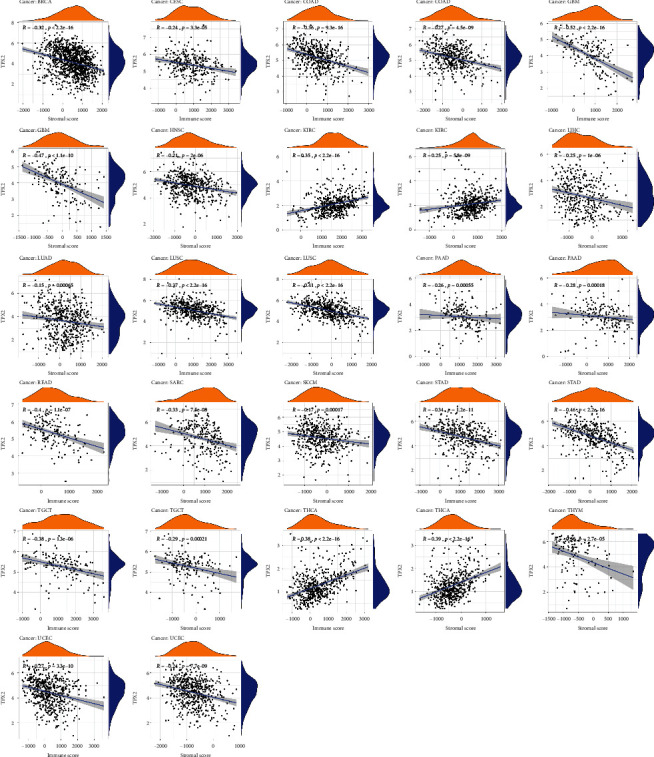
Correlation analysis of TPX2 expression levels with stromal and immune scores in 18 cancer types.

**Figure 7:**
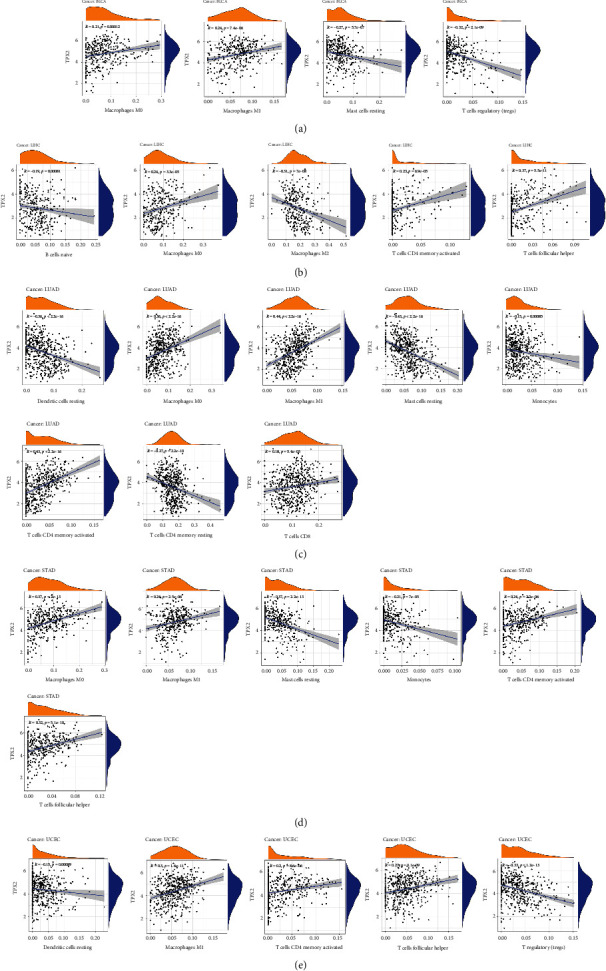
Correlation analysis of TPX2 expression levels with the level of immune cell infiltration in BLCA (a), LIHC (b), LUAD (c), STAD (d), and UCEC (e) across cancer types.

Figure 6 illustrates the cancers with the most significant relationship between TPX2 expression and stromal scores, which were GBM, STAD, LUSC, THCA, and THYM, whereas the cancers with the most significant relationship between TPX2 expression and the immune score included GBM, READ, TGCT, THCA, and LUSC. We also evaluated whether TPX2 expression is related to the infiltration of immune cells in several cancers. In BLCA, LIHC, LUAD, STAD, and UCEC, TPX2 expression was significantly associated with infiltrating immune cells, as shown in Figure 7. In diverse cancer types, considerable coexpression of TPX2 with immune checkpoint genes, including *CTLA4*, *LAG3*, *TIGIT*, *PDCD1*, *CD27*, *CD28*, *CD40*, *CD44*, *CD48*, *CD70*, *CD80*, *CD86*, *CD160*, *CD200*, *CD244*, *CD274*, and *CD276*, was found. These findings suggested that TPX2 is involved in the regulation of the tumor immune response via immune checkpoint activity modulation (Figure S1).

### 3.6. GSEA

**Figure 8:**
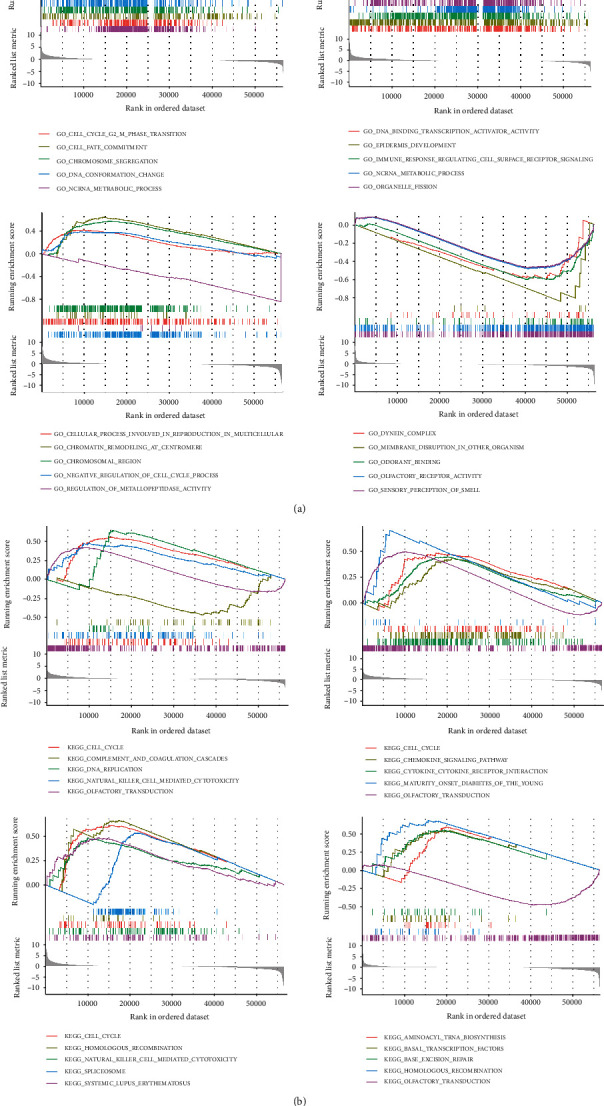
Gene set enrichment analysis (GSEA) of TPX2. (a) Top five results of TPX2 GSEA ranked according to their correlation with biological processes in BRCA, KIRC, LUAD, and UCEC. (b) Top five results of TPX2 GSEA ranked according to their correlation with signaling pathways in BRCA, KIRC, LUAD, and UCEC.

We next divided the cancer samples into two groups based on high or low TPX2 expression levels and used GSEA to identify the enrichment of GO and KEGG gene sets in the two groups.  Figure 8(a) shows the biological processes that were highly enriched in both groups. G2-M phase transition, DNA-binding transcription activator activity, cellular processes involved in reproduction in multicellular organisms, and dynein complexes were found to be the most enriched biological processes in BRCA, KIRC, LUAD, and UCEC, respectively.  Figure 8(b) also shows the signaling pathways that were highly enriched in both groups. Cell cycle signaling pathways were the most enriched pathways in BRCA, KIRC, and LUAD, whereas aminoacyl tRNA biosynthesis was the most enriched in UCEC. The results further showed that TPX2 is mainly involved in the cell cycle regulation signaling pathway. We found that TPX2 expression is highly correlated with cell cycle biological processes and also analyzed the correlation among TPX2 expression levels, cell cycle regulators (CCNA2, Cyclin A2; CDK2, Cyclin-dependent kinase 2), and glycolytic metabolic pathway key molecules (HK2, PFKM, and PKM) using the GEPIA database (http://gepia.cancer-pku.cn/index.html). The results showed that the expression of TPX2 was significantly positively correlated with the expression of cell cycle regulators (CCNA2 and CDK2) in BRCA, KIRC, LUAD, and UCEC (Figure S2) and that the expression of TPX2 was significantly positively correlated with the expression of glycolytic metabolic pathway key molecules (HK2, PFKM, and PKM) in LUAD and UCEC (Figure S3).

## 4. Discussion

TPX2 is a microtubule-associated protein that directs the kinesin Xklp2 to mitotic spindle poles [20, 21], and it plays a vital role in the formation of the microtubules that make up the spindle [22]. TPX2 is overexpressed in esophageal cancer, colorectal cancer, hepatocellular carcinoma, colon cancer, bladder cancer, clear cell renal carcinoma, pancreatic cancer, ovarian carcinoma, breast cancer, and neuroblastoma, and its degree of expression has been associated with poor prognoses [23–32]. Combined with its critical role as a mitotic regulator, this association implicates TPX2 as a potential oncogene. In this study, we discovered a correlation between TPX2 overexpression and poor prognosis among most cancers. However, it is unclear how TPX2 overexpression contributes to genomic instability and carcinogenesis. This might contribute to carcinogenesis by causing spindle malfunction and chromosomal instability.

Several studies have found that a reduction in TPX2 levels is beneficial for cancer treatment. For example, the depletion of TPX2 can significantly inhibit prostate cancer and cholangiocarcinoma cell activity and migration, and TPX2 knockdown can inhibit tumor growth considerably *in vivo* [33–35]. Furthermore, the upregulation of TPX2 expression has been shown to significantly promote non-small-cell lung cancer, hepatocellular cell migration, and invasion; it has also been associated with increased cell plasticity [36, 37]. TPX2 siRNA causes apoptosis, decreased cell proliferation, and invasion. TPX2 has been shown to play a role in tumor growth regulation in cervical cancer, hepatocellular carcinoma, and glioma [38–40]. Further, TPX2 siRNA inhibits tumor cell invasion and metastasis promotes tumor cell death, and could be a potential treatment option for esophageal carcinoma, medullary thyroid carcinoma, colon cancer, and breast cancer [41–44]. Our findings suggest a correlation between TPX2 overexpression and poor prognosis among most cancers and the potential for it to be used as an important target in antitumor metastasis therapy, which is conducive to precision medicine, for most malignancies.

We also investigated the relationship between TPX2 expression and TMB/MSI. This relationship was statistically significant in the majority of cancer types, with THYM (TMB) and SARC (MSI) having the highest correlation coefficients. We also analyzed the correlation between TPX2 expression levels and the tumor microenvironment and immune cell infiltration. In most cancer types, TPX2 expression levels were found to be negatively correlated with stromal and immune cell contents, but the opposite was true for KIRC and THCA. Meanwhile, our data indicate that TPX2 is involved in the recruitment and modulation of tumor-immune infiltrating cells, and that for BLCA, LIHC, LUAD, STAD, and UCEC, it might be employed as a predictive biomarker. Therefore, in the future, on one hand, we can estimate the effect of immunotherapy by detecting the expression level of TPX2, and on the other hand, we can develop targeted therapy for TPX2 for combinations with traditional immunotherapy to improve its efficacy.

The process through which TPX2 depletion causes cancer cells to die is unclear; however, it might involve mitotic disruption. TPX2 expression has been found to be increased in ovarian cancer tissues, and knocking it out suppresses the expression of polo-like kinase 1. Since this kinase regulates the M phase of the cell cycle and the activity of Cdc2, its suppression results in cell arrest during the G2/M phase checkpoint and, therefore decreased cancer proliferation [45]. After hnRNP-F knockdown, TPX2 levels were found to decline even further, causing cyclin D1 protein expression to decrease and p21 protein expression to increase, resulting in cell cycle arrest and the reduced proliferation of bladder cancer cells [46]. Our GSEA results revealed that TPX2 gene activity is linked to cell cycle factors (CCNA2 and CDK2) and that the TPX2 expression level is positively correlated with the expression of CCNA2 and CDK2 in BRCA, KIRC, LUAD, and UCEC. TPX2 is mainly involved in cell cycle regulation and promotes tumorigenesis and development. In addition, TPX2 expression was determined to be positively related to key molecules of the glycolysis metabolic pathway, and it might be involved in this pathway. In this study, we also found that TPX2 expression is highly correlated with multiple checkpoint molecules in multiple cancer types. This suggested potential synergy between TPX2 and known immune checkpoints. Finally, our findings suggest that TPX2 has a carcinogenic effect in many cancers and is a promising potential cancer treatment target. The limitation of this study is that we used a bioinformatic approach, and thus, further biological experiments are needed to validate these claims.

## 5. Conclusions

In this study, we performed GSEA of TPX2 and comprehensively analyzed its association with prognosis, TMB, MSI, tumor microenvironments, and immune cell infiltration in 33 cancer types through an extensive bioinformatic pan-cancer analysis. Our findings suggest that TPX2 has a carcinogenic effect on a variety of cancers and that it could be a marker of immune infiltration and poor prognosis. While the mechanism by which TPX2 overexpression leads to cancer remains unclear, it likely involves the role of this protein in the regulation of mitotic spindle microtubules. We proposed that TPX2 can be used as a prognostic biomarker and therapeutic target for a variety of cancers.

## Data Availability

The data included in the current study are available in the TCGA database (https://cancergenome.nih.gov/). The data used to support the findings of this study are included within the article.

## Supplementary Materials

Supplementary Materials Table S1: full names, abbreviations, and gene expression data associated with samples of 33 cancer types. Figure S1: TPX2 expression is strongly correlated with immune checkpoint gene expression in most cancer types (excluding ACC, CHOL, DLBC, OV, PCPG, and UVM). ^∗^represents *p* < 0.05, ^∗∗^represents *p* < 0.01, and ^∗∗∗^represents *p* < 0.001. Figure S2: the expression of TPX2 is significantly positively correlated with the expression of cell cycle regulators (CCNA2 and CDK2) in BRCA, KIRC, LUAD, and UCEC. Figure S3: the expression of TPX2 is significantly positively correlated with the expression of glycolytic metabolic pathway key molecules (HK2, PFKM, and PKM) in LUAD and UCEC.
