# Supplementary material for: Comprehensive Analysis of the Oncogenic Role of Targeting Protein for Xklp2 (TPX2) in Human Malignancies
Source: Dis Markers. 2022 Oct 18;2022:7571066. doi: 10.1155/2022/7571066 (PMC9596273; doi:10.1155/2022/7571066)
Supplement: Supplementary Materials — Table S1: full names, abbreviations, and gene expression data associated with samples of 33 cancer types. Figure S1: TPX2 expression is strongly correlated with immune checkpoint gene expression in most cancer types (excluding ACC, CHOL, DLBC, OV, PCPG, and UVM). ∗represents p < 0.05, ∗∗represents p < 0.01, and ∗∗∗represents p < 0.001. Figure S2: the expression of TPX2 is significantly positively correlated with the expression of cell cycle regulators (CCNA2 and CDK2) in BRCA, KIRC, LUAD, and UCEC. Figure S3: the expression of TPX2 is significantly positively correlated with the expression of glycolytic metabolic pathway key molecules (HK2, PFKM, and PKM) in LUAD and UCEC. [file 7571066.f1.docx]

Table S1: Full names, abbreviations, and gene expression data samples of 33 cancer types.

| Cancer Types | abbreviation | Number of Samples | | |
| --- | --- | --- | --- | --- |
|  |  | total | cancer | normal |
| Adrenocortical carcinoma | ACC | 79 | 79 | 0 |
| Bladder urothelial carcinoma | BLCA | 430 | 411 | 19 |
| Breast invasive carcinoma | BRCA | 1217 | 1104 | 113 |
| Cervical squamous cell carcinoma and endocervical adenocarcinoma | CESC | 309 | 306 | 3 |
| Cholangiocarcinoma | CHOL | 45 | 36 | 9 |
| Colon adenocarcinoma | COAD | 512 | 471 | 41 |
| Lymphoid neoplasm diffuse large B-cell lymphoma | DLBC | 48 | 48 | 0 |
| Esophageal carcinoma | ESCA | 173 | 162 | 11 |
| Glioblastoma multiforme | GBM | 173 | 168 | 5 |
| Head and Neck squamous cell carcinoma | HNSC | 546 | 502 | 44 |
| Kidney chromophobe | KICH | 89 | 65 | 24 |
| Kidney renal clear cell carcinoma | KIRC | 607 | 535 | 72 |
| Kidney renal papillary cell carcinoma | KIRP | 321 | 289 | 32 |
| Acute Myeloid Leukemia | LAML | 151 | 151 | 0 |
| Brain lower grade glioma | LGG | 529 | 529 | 0 |
| Liver hepatocellular carcinoma | LIHC | 424 | 374 | 50 |
| Lung adenocarcinoma | LUAD | 585 | 526 | 59 |
| Lung squamous cell carcinoma | LUSC | 550 | 501 | 49 |
| Mesothelioma | MESO | 86 | 86 | 0 |
| Ovarian serous cystadenocarcinoma | OV | 379 | 379 | 0 |
| Pancreatic adenocarcinoma | PAAD | 182 | 178 | 4 |
| Pheochromocytoma and Paraganglioma | PCPG | 186 | 183 | 3 |
| Prostate adenocarcinoma | PRAD | 551 | 499 | 52 |
| Rectum adenocarcinoma | READ | 177 | 167 | 10 |
| Sarcoma | SARC | 265 | 263 | 2 |
| Skin cutaneous melanoma | SKCM | 472 | 471 | 1 |
| Stomach adenocarcinoma | STAD | 407 | 375 | 32 |
| Testicular germ cell tumors | TGCT | 156 | 156 | 0 |
| Thyroid carcinoma | THCA | 568 | 510 | 58 |
| Thymoma | THYM | 121 | 119 | 2 |
| Uterine corpus endometrial carcinoma | UCEC | 583 | 548 | 35 |
| Uterine carcinosarcoma | UCS | 56 | 56 | 0 |
| Uveal melanoma | UVM | 80 | 80 | 0 |


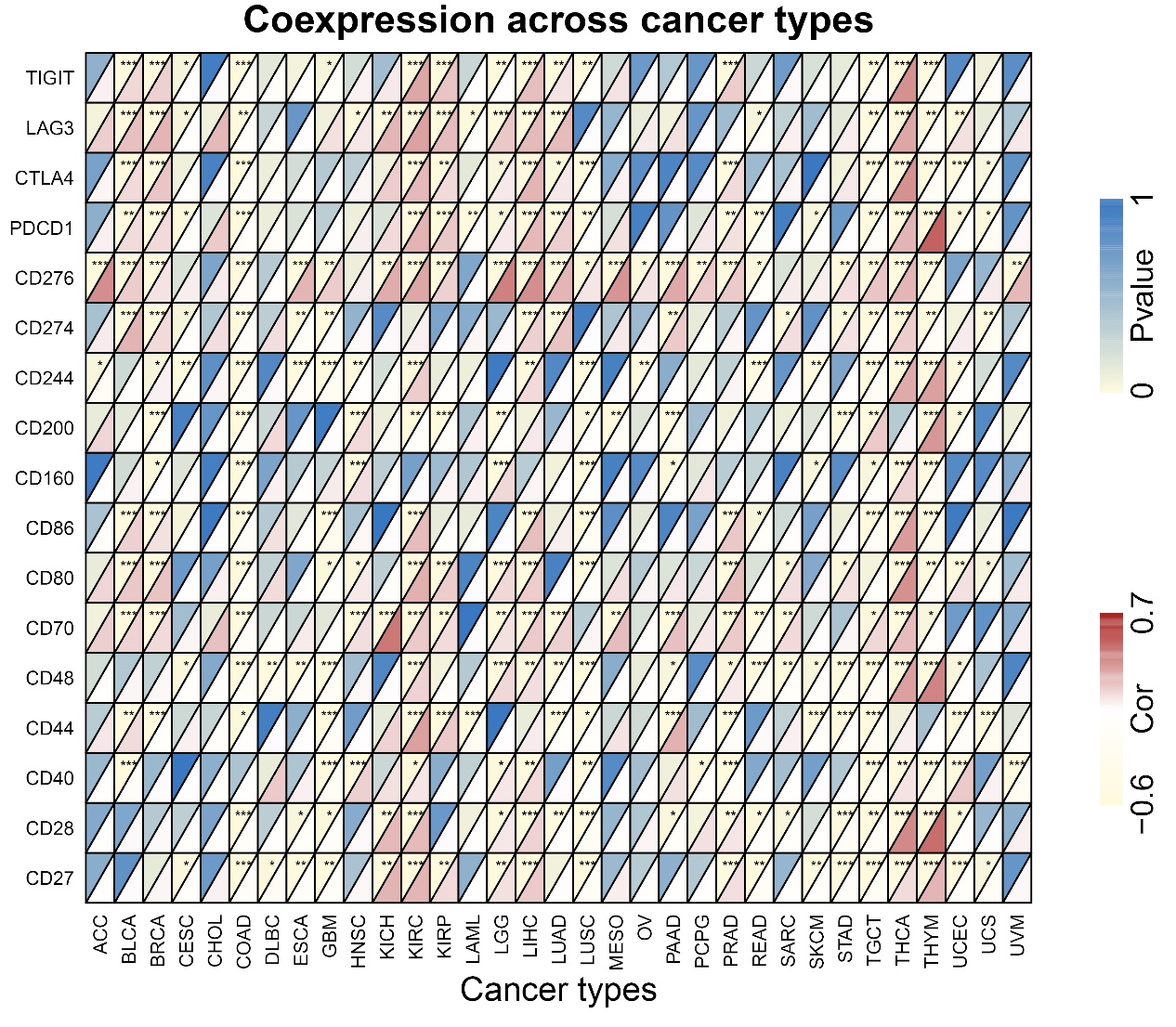


FIGURE S1: TPX2 expression is strongly correlated with immune checkpoint gene expression in most cancer types (excluding ACC, CHOL, DLBC, OV, PCPG, and UVM). * represents *p* < 0.05, ** represents *p* < 0.01, and *** represents *p* < 0.001.


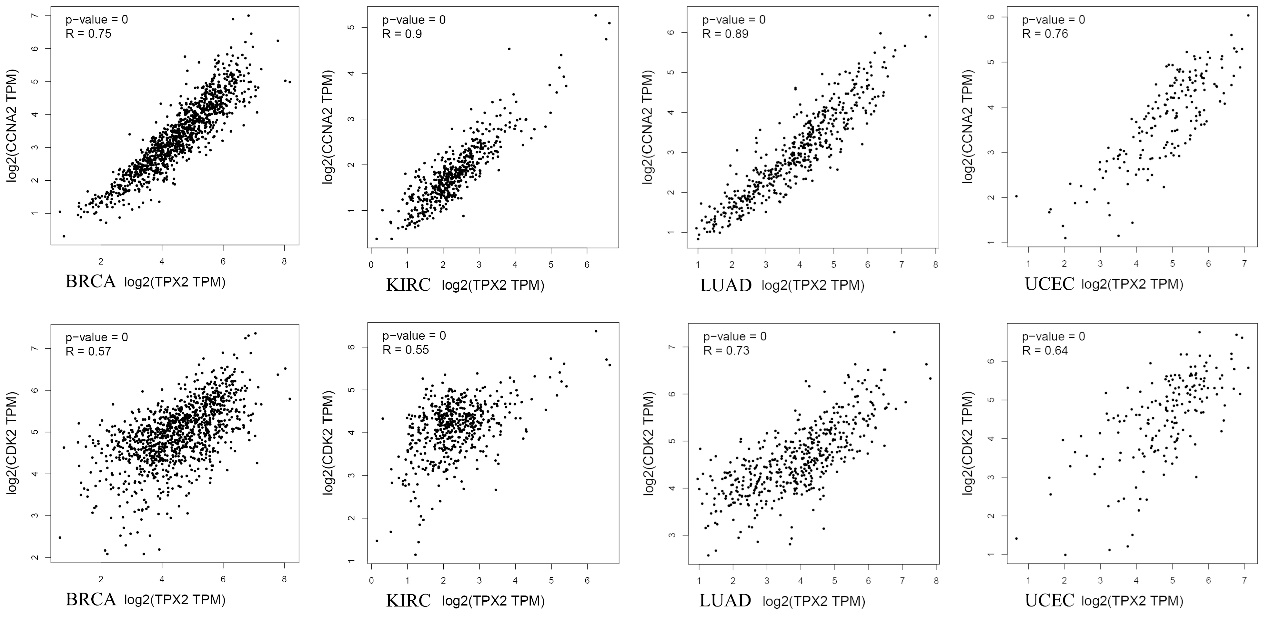


FIGURE S2: The expression of TPX2 is significantly positively correlated with the expression of cell cycle regulators (CCNA2, CDK2) in BRCA, KIRC, LUAD, and UCEC.


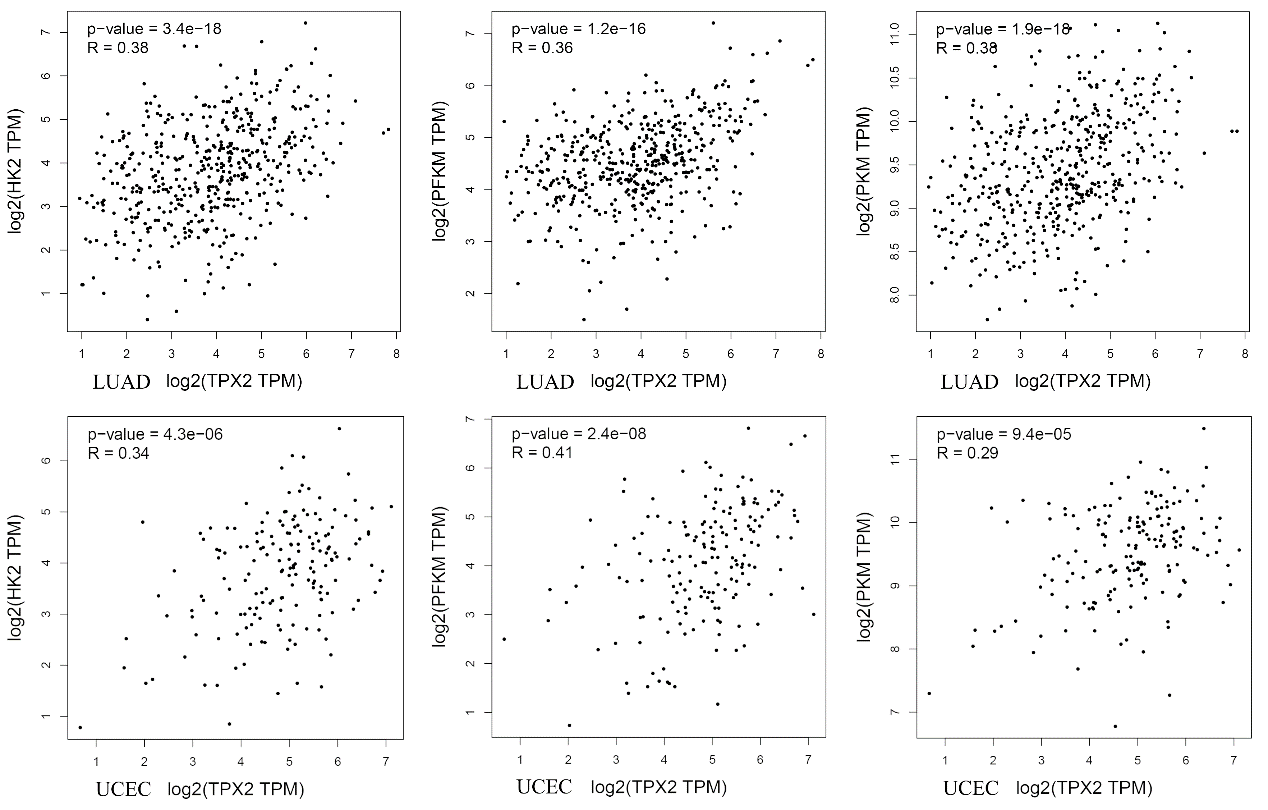


FIGURE S3: The expression of TPX2 is significantly positively correlated with the expression of glycolytic metabolic pathway key molecules (HK2, PFKM, and PKM) in LUAD and UCEC.
